# The clinical and molecular genetic study of 20 Silver Russell Syndrome cases

**DOI:** 10.1186/1687-9856-2013-S1-P45

**Published:** 2013-10-03

**Authors:** Chunxiu Gong, Ming-Qiang Zhu, Di Wu, Bingyan Cao

**Affiliations:** 1 Beijing Children's Hospital, The Capital University, Beijing, China

## Objective

To analyze the genetic pathogenesis and improve the accuracy of the diagnosis of the disease, this study reported the clinical features of 20 patients with Silver Russell Syndrome (SRS) in the Beijing Children's Hospital and detected the chromosome 11p15 imprinting defects in 16 patients of them.

## Methods

20 SRS cases diagnosed in Beijing Children’s Hospital from 2006 to 2011 were studied retrospectively for clinical manifestations, physical signs, laboratory examinations and respond of GH treatment. We compared with 3 different diagnostic criteria and used the methylation-specific multiplex ligation dependent probe amplification (MS-MLPA) method to detect the chromosome 11p15 imprinting defects in 16 patients of them, meanwhile take 10 normal control.

## Results

We collected 20 SRS patients over a period from 2006 to 2011 with 3 criteria. The concordance is 90%. Include fifteen males and five females, age range 0.08~12.17yr. The most chief complaint is short, 85%. Then, it is asymmetry (5%), and external genital abnormalities (10%). The clinical characteristics with the frequencies accounted for over 80% included small for gestation age(SGA), postnatal growth retardation, craniofacial dysmorphism, asymmetry and super thin of whole body, extremely limbs, fifth finger clinodactyly, BMI<-2SDS and height was obviously lagging behind the bone age. Six of them had used the growth hormone for 3-months to 12 Months. The growth velocity is from 4cm to 10cm/year. In the 16 patients, 6 patients were found hypomethylation in chromosome 11p15 ICR1, the other one with chromosome 11p15 ICR1 hypomethylation and ICR2 hypermethylation may be the result of the maternal chromosome 11p15 uniparental disomy. Another case had duplication of the maternal chromosome 11p15 fragment. Two cases had good effects of GH treatment, one with chromosome 11p15 ICR1 hypomethylation and the other one was normal.

## Conclusion

The top 3 clinical features in SRS are ① growth retardation include SGA and/or postnatal. ② Malformation include craniofacial dysmorphism, asymmetry of face and/or limbs, fifth finger clinodactyly. ③ Super severe low BMI and height was obviously lagging behind bone age. No laboratory and imagination specificity. Chromosome 11p15 imprinting defect is the major genetic disturbance in SRS, about 50%, and ICR1 hypomethylation is the predominant molecular alteration. The MS-MLPA is a technique for detecting all chromosome 11p15 imprinting defects of SRS. It is useful for the study of the genetic aspects of SRS. The relation between the respond of GH treatment and genetic changes is uncertainty.

